# Life Cycle Environmental Impacts of Electricity from Biogas Produced by Anaerobic Digestion

**DOI:** 10.3389/fbioe.2016.00026

**Published:** 2016-03-11

**Authors:** Alessandra Fusi, Jacopo Bacenetti, Marco Fiala, Adisa Azapagic

**Affiliations:** ^1^Sustainable Industrial Systems, School of Chemical Engineering and Analytical Science, The University of Manchester, Manchester, UK; ^2^Dipartimento di Scienze Agrarie e Ambientali – Produzione, Territorio, Agroenergia, Università degli Studi di Milano, Milan, Italy

**Keywords:** agricultural waste, anaerobic digestion, biogas, electricity, life cycle assessment, renewable energy

## Abstract

The aim of this study was to evaluate life cycle environmental impacts associated with the generation of electricity from biogas produced by the anaerobic digestion (AD) of agricultural products and waste. Five real plants in Italy were considered, using maize silage, slurry, and tomato waste as feedstocks and cogenerating electricity and heat; the latter is not utilized. The results suggest that maize silage and the operation of anaerobic digesters, including open storage of digestate, are the main contributors to the impacts of biogas electricity. The system that uses animal slurry is the best option, except for the marine and terrestrial ecotoxicity. The results also suggest that it is environmentally better to have smaller plants using slurry and waste rather than bigger installations, which require maize silage to operate efficiently. Electricity from biogas is environmentally more sustainable than grid electricity for seven out of 11 impacts considered. However, in comparison with natural gas, biogas electricity is worse for seven out of 11 impacts. It also has mostly higher impacts than other renewables, with a few exceptions, notably solar photovoltaics. Thus, for the AD systems and mesophilic operating conditions considered in this study, biogas electricity can help reduce greenhouse gas (GHG) emissions relative to a fossil-intensive electricity mix; however, some other impacts increase. If mitigation of climate change is the main aim, other renewables have a greater potential to reduce GHG emissions. If, in addition to this, other impacts are considered, then hydro, wind, and geothermal power are better alternatives to biogas electricity. However, utilization of heat would improve significantly its environmental sustainability, particularly global warming potential, summer smog, and the depletion of abiotic resources and the ozone layer. Further improvements can be achieved by banning open digestate storage to prevent methane emissions and regulating digestate spreading onto land to minimize emissions of ammonia and related environmental impacts.

## Introduction

The need to mitigate climate change and improve security of energy supply is driving a growing interest in renewable energy sources, with many world regions and countries setting ambitious targets. For example, the EU directive on the promotion of the use of energy from renewable sources (EC, 2009) sets the target of achieving a 20% share of energy from renewable resources by 2020, including biogas produced by anaerobic digestion (AD) of agricultural feedstocks.

Production of biogas is expanding rapidly in Europe. According to EurObserv’ER (2014), about 13.4 million ton oil equivalent (Mtoe) of biogas primary energy was produced in the EU during 2013, a 10% increase on the 2012 levels. Germany is the largest producer of biogas, not only in Europe but also in the world. In 2013, it had 7874 AD plants with a total installed electrical capacity of 3384 MW, which generated 27 TWh/year (EurObserv’ER, 2014; Fuchsz and Kohlheb, 2015). By comparison, the second largest world producer – China – generates just over one-quarter of that (7.6 TWh/year in 2009) (Chen et al., 2012). Italy follows closely in third place at 7.4 TWh of electricity per year produced by 1300 AD plants with a total installed capacity of 1000 MW (Brizzo, 2015). The plants are fed largely with maize grown specifically for this purpose, which in Italy occupies 10% of the total maize cultivation area (1,172,000 ha) (Casati, 2011). However, this is still only half the area in Germany (2,282,000 ha) where it covers one-third of the total maize land (Dressler et al., 2012).

The rapid expansion of biogas production in Europe is largely due to the feed-in-tariffs (FiT) schemes available in 29 countries (Whiting and Azapagic, 2014). For example, electricity generators in Italy using biogas produced in AD plants smaller than 1 MW are paid €280/MWh generated. In the UK, the subsidies are significantly lower, ranging from €130 to 210/MWh, depending on the plant size (Whiting and Azapagic, 2014). This perhaps explains why the deployment of AD was initially slower than in Italy, with only 180 AD plants installed so far, but with a further 500 projects currently under development (NNFCC, 2015). However, the FiT scheme in Italy has recently been changed, reducing the subsidy for electricity by 15–30% and introducing payments for utilization of heat and other coproducts (Ministero dello Sviluppo Economico, 2012). In the US, the growth of biogas production has also been slower than elsewhere, with only 244 AD plants currently in operation (Ebner et al., 2015); this is largely due to the absence of adequate subsidies.

Biogas produced by AD is considered to have a high saving potential with respect to greenhouse gas (GHG) emissions (EC, 2009). However, beyond that, other environmental implications of biogas production are still unclear despite quite a few life cycle assessment (LCA) studies having been carried out. This is due to several reasons. First, most previous studies of biogas have either focused on climate change or considered a limited number of impacts; for a summary, see Table 1. As far as the authors are aware, out of 26 studies found in the literature, only five have considered a full suite of impacts normally included in LCA studies, two of which are based in the UK (Mezzullo et al., 2013; Whiting and Azapagic, 2014), one in Argentina (Morero et al., 2015), one in Italy (Pacetti et al., 2015), and one in China (Xu et al., 2015). It is also apparent from Table 1 that the goal, scope, life cycle impact assessment (LCIA) methodology, feedstocks, and geographical regions covered by the studies vary widely. Most studies are based in Europe with several in China and one each in Argentina, Canada, and the US. All plants have a capacity below 1 MW, with the majority being around 500 kW (where reported); some are electricity only and others combined heat and power (CHP) installations. Most studies have excluded the impacts of constructing and decommissioning the AD and power plants. Maize is the most commonly considered feedstock, followed by animal slurry. The functional unit is largely based either on a unit of feedstock used to generate biogas or a unit of energy (biogas, heat, or electricity). Most studies have relied on secondary foreground data to estimate the impacts or used only limited primary data. However, the greatest variation among the studies is found in the number of impacts considered and the methodologies used to estimate them. The former range from 1 to 18 and the latter cover almost all known LCIA methods, including EcoIndicator 99 (Goedkoop and Spriensma, 2001), CML 2001 (Guinée et al., 2002), Impact 2002+ (Olivier et al., 2003), and ReCiPe (Goedkoop et al., 2009). These and the other differences, including the credits for coproducts, have led to very different results among the studies, making it difficult to compare them, and draw any generic conclusions on the environmental sustainability of biogas.

**Table 1:** **LCA biogas studies available in the literature**.

Reference	Country	No. of AD plants	Plant size	Feedstocks^a^	Functional unit	Foreground LCI data^b^	Capital goods	Impacts (LCIA method)^c^	Best options^c^
Jury et al. (2010)	Luxemburg	Not reported	Not reported	• 4 winter cereals • 4 summer cereals	1 MJ supplied to the natural gas grid	Secondary	Excluded	GWP and CED (impact 2002+)	Not reported
De Vries et al. (2010)	Western Europe	Not reported	Not reported	• Cattle slurry • Maize silage • Codigestion of above	1 ton of feedstock (wet)	Secondary	Excluded	GWP, AP, EP, CED, and LU (not specified)	Codigestion for GWP, EP, AP, and CED; slurry for LU
Blengini et al. (2011)	Italy	Not reported	Not reported	• Maize • Sorghum • Triticale • Miscanthus • Slurry	1 MJ of net energy (heat or electricity) delivered	Secondary	Included	6 (CML 2001)	Miscanthus for GWP, EP, and AP; maize silage for photochemical smog
Dressler et al. (2012)	Germany	1	510 kW	• Maize silage	1 kWh of electricity	Secondary	Excluded	GWP, AP, EP (CML 2001)	Not reported
Lansche and Müller (2012)	Germany	1	186 kW	• Cattle slurry • Maize silage • Grass silage • Codigestion of above	1 MJ of electricity	Primary	Excluded	GWP, AP, EP (CML 2001)	Cattle slurry
Meyer-Aurich et al. (2012)	Germany	1	500 kW	• Cattle slurry • Maize silage • Codigestion of above	1 kWh of electricity	Secondary	Excluded	GWP (IPCC, 2007)	Cattle slurry
De Vries et al. (2012)	The Netherlands	1	500 kW	• Pig slurry • Maize silage • Glycerine • Beet tails • Roadside grass • Codigestion of above	1 ton of feedstock (wet)	Secondary	Excluded	7 (ReCiPe mid-point)	Pig slurry for GWP, AP, ME, and LU; codigestion for FFD, FE, and PMF
Bacenetti et al. (2013)	Italy	3	250–999 kW	• Maize silage • Pig slurry • Codigestion of above	1 kWh of electricity	Primary	Excluded	GWP and CED (IPCC, 2007)	Pig slurry for GWP; maize silage for CED
Mezzullo et al. (2013)	UK	1	Not reported	• Cattle slurry	1 m^3^ of methane	Secondary	Included	11 (Ecoindicator 99)	Not reported
Zhang et al. (2013)	China	1	Not reported	• Household waste	Household biogas (digester volume 8 m^3^)	Secondary	Included	CO_2_ emissions (Not specified)	Not reported
Lijó et al. (2014a)	Italy	2	250 and 500 kW	• Animal slurry • Maize silage	1 ton of feedstock (wet)	Primary only for AD and CHP plant	Excluded	8 (ReCiPe mid-point)	Animal slurry
Lijó et al. (2014b)	Italy	1	500 kW	• Codigestion of maize and triticale silage	100 kWh of electricity	Primary only for AD and CHP plant	Excluded	8 (ReCiPe mid-point)	Maize silage
Rodriguez-Verde et al. (2014)	Spain	1	500 kW	• Pig slurry • Molasses • Fish • Biodiesel • Vinasse residues	110,000 ton/year of pig slurry	Primary and secondary	Excluded	6 (CML 2001)	Not reported
Styles et al. (2014)	UK	4	72–185 kW	• Food waste • Cattle slurry • Maize and grass silage • Miscanthus • Codigestion of above	1 year of farm operation	Secondary	Excluded	GWP, AP, EP, and RDP (CML 2010)	Slurry and food waste
Whiting and Azapagic (2014)	UK	1	170 kW	• Codigestion of slurry, cheese whey, fodder beet, and maize silage	Cogeneration of 1 MWh of heat and electricity	Primary and secondary	Included	11 (CML 2001)	Farm waste better than maize for 8 out of 11 impacts
Bacenetti and Fiala (2015)	Italy	5	100–999 kW	• Cattle slurry • Pig slurry • Cereal silage • Codigestion of above	1 kWh of electricity		Tractors and equipment included; AD and CHP plant excluded	GWP (IPCC, 2007)	Feedstocks
Ebner et al. (2015)	USA	1	Not reported	• Codigestion of cattle slurry and food waste	1 ton of feedstock (wet)	Secondary	Excluded	GWP (IPCC, 2007)	Not reported
Fuchsz and Kohlheb (2015)	Germany	3	600 kW	• Maize silage • Cow slurry • Codigestion of above	1 kWh of electricity	Primary only for AD plant construction	Included	GWP, AP, EP (not specified)	Maize silage for GWP; slurry for AP and EP
Ingrao et al. (2015)	Italy	1	999 kW	• Codigestion of by-products from wheat processing and maize silage	1 kWh of electricity	Primary	Excluded	GWP (IPCC, 2007)	Not reported
Jin et al. (2015)	China	1	Not reported	• Food waste	1 ton of food waste	Secondary	Excluded	5 (CML 2001)	Not reported
Lijó et al. (2015)	Italy	1	1000 kW	• Codigestion of pig slurry and maize silage	1 ton of feedstock (wet)	Primary only for AD and CHP plant	Excluded	8 (ReCiPe mid-point)	Not reported
Morero et al. (2015)	Argentina	2	531–573 kW	• Agroindustrial wastes	1 m^3^ of biogas and 1 kWh of electricity	Primary and secondary	Excluded	11 (CML 2001)	Not reported
Pacetti et al. (2015)	Italy	1	Not reported	• Maize • Sorghum • Wheat silage	1 GJ of energy in the biogas	Secondary	Excluded	18 (ReCiPe mid-point)	Sorghum
Siduo et al. (2015)	Canada	Not reported	Not reported	• Dairy slurry	1100 ton of dairy slurry	Primary and secondary	Excluded	7 (CML 2001)	Not reported
Xu et al. (2015)	China	Not reported	Not reported	• Food waste	1 ton of volatile solids	Secondary	Excluded	18 (ReCiPe mid-point)	Not reported
This study	Italy	5	100–999 kW	• Maize silage • Cow slurry • Codigestion of pig slurry, tomato waste, and maize silage • Codigestion of pig slurry and maize silage • Codigestion of pig slurry, maize silage, and maize ear silage	1 kWh of electricity	Primary	Included	11 (CML 2001)	Slurry for 9 out of 11 impacts; codigestion of slurry, waste, and maize sludge for marine and terrestrial ecotoxicity

*^a^Each bullet point represents a feedstock stream fed to the anaerobic digesters one at a time*.

*^b^LCI, life cycle inventory. Foreground data refer to the AD and CHP plants. Primary data are directly measured and/or collected as part of the study. Secondary data are from databases and literature*.

*^c^LCIA, life cycle impact assessment; AP, acidification potential; CED, cumulative energy demand; EP, eutrophication potential; FE, fresh water eutrophication; FFD, fossil fuel depletion; GWP, global warming potential; LU, land use; ME, marine eutrophication; PMF, particulate matter formation; RDP, resource depletion potential*.

This study aims to make further contributions to the discussion on the environmental sustainability of biogas. The paper considers life cycle environmental impacts of electricity generation in five real AD-CHP systems using biogas produced from differing mixes of four types of feedstock. The plants are situated in Italy. The novel aspects of the work compared to previous studies include:

- estimation of impacts associated with electricity generated from biogas using different feedstocks, including dedicated maize crops, their mixture with animal slurry, and agricultural waste as well as a mixture of slurry and waste;
- use of primary data for both the feedstock production and operation of the AD-CHP systems;
- consideration of the influence of different scales of the AD-CHP systems on the environmental impacts;
- inclusion of construction and decommissioning of AD and CHP plants;
- estimation of the avoided emissions from using the digestate instead of slurry as fertilizer; and
- comparison of impacts with grid electricity, natural gas, and renewable sources of electricity.

## Materials and Methods

The environmental impacts of biogas electricity were estimated using LCA as a tool. The study was carried out in accordance with the ISO 14040/44 methodology for LCA (ISO, 2006a,b). The systems were modeled using Gabi LCA software V6.11 (Thinkstep, 2015). The CML 2001 method (Guinée et al., 2002), April 2013 update, was followed to estimate the following 11 impacts considered in this method: abiotic depletion potential of elements (ADP elements), abiotic depletion potential of fossil fuels (ADP fossil), acidification potential (AP), eutrophication potential (EP), freshwater aquatic ecotoxicity potential (FAETP), global warming potential (GWP), human toxicity potential (HTP), marine aquatic ecotoxicity potential (MAETP), ozone layer depletion potential (ODP), photochemical oxidants creation potential (POCP), also known as summer smog, and terrestrial ecotoxicity potential (TETP). For further details on the estimation of the impacts, see Supplementary Material.

The next sections detail the goal of the study, the assumptions, and data used in the study.

### Goal and Scope of the Study

The main goal of the study was to estimate the environmental impacts of electricity generated by different AD-CHP systems utilizing maize silage and agricultural waste. The results were compared with electricity from the grid, natural gas, and different renewables to help evaluate the environmental sustainability of biogas electricity relative to other available options.

Five real AD-CHP systems were considered using differing combinations of the following feedstocks: maize and maize ear silage; pig and cow slurry; and tomato peel and seeds (Table 2). The volume of the AD digesters ranged from 1650 to 2750 m^3^ and the installed electrical capacity of the CHP plants from 100 to 999 kW. The plants are located at farms producing the feedstocks in Lombardy in Northern Italy, where the majority of the country’s biogas plants are situated (Negri et al., 2014).

**Table 2:** **Summary of the main characteristics of the AD-CHP plants considered in the study**.^a^

Plant	Feedstock	Volume of AD digesters (m^3^)	Dry matter content in digesters (%)	Organic loading in digesters (kg/day⋅m^3^)	Methane content in biogas (%)	Installed CHP power (kW)	Electricity generation (MWh/year)	Electricity consumption (MWh/year)	Heat generation (MWh/year)	Heat consumption by AD (MWh/year)
Plant 1	• Pig slurry • Tomato peel and seeds • Maize silage	1650	8.7	0.92	52.8	230	1945	173	2549	809
Plant 2	• Pig slurry • Maize silage	2250	10.6	1.07	52.6	300	2429	206	3184	814
Plant 3	• Pig slurry • Maize silage • Maize ear silage	2000	9.7	0.98	52.7	300	2505	276	3514	799
Plant 4	• Maize silage	2 × 2750	10.7	3.40	52.1	999	7972	717	8771	2505
Plant 5	• Cow slurry	1850	8.5	0.58	56.0	100	781	86	1095	547

*^a^All data sourced directly from the farm/plant owners*.

**Figure 1:**
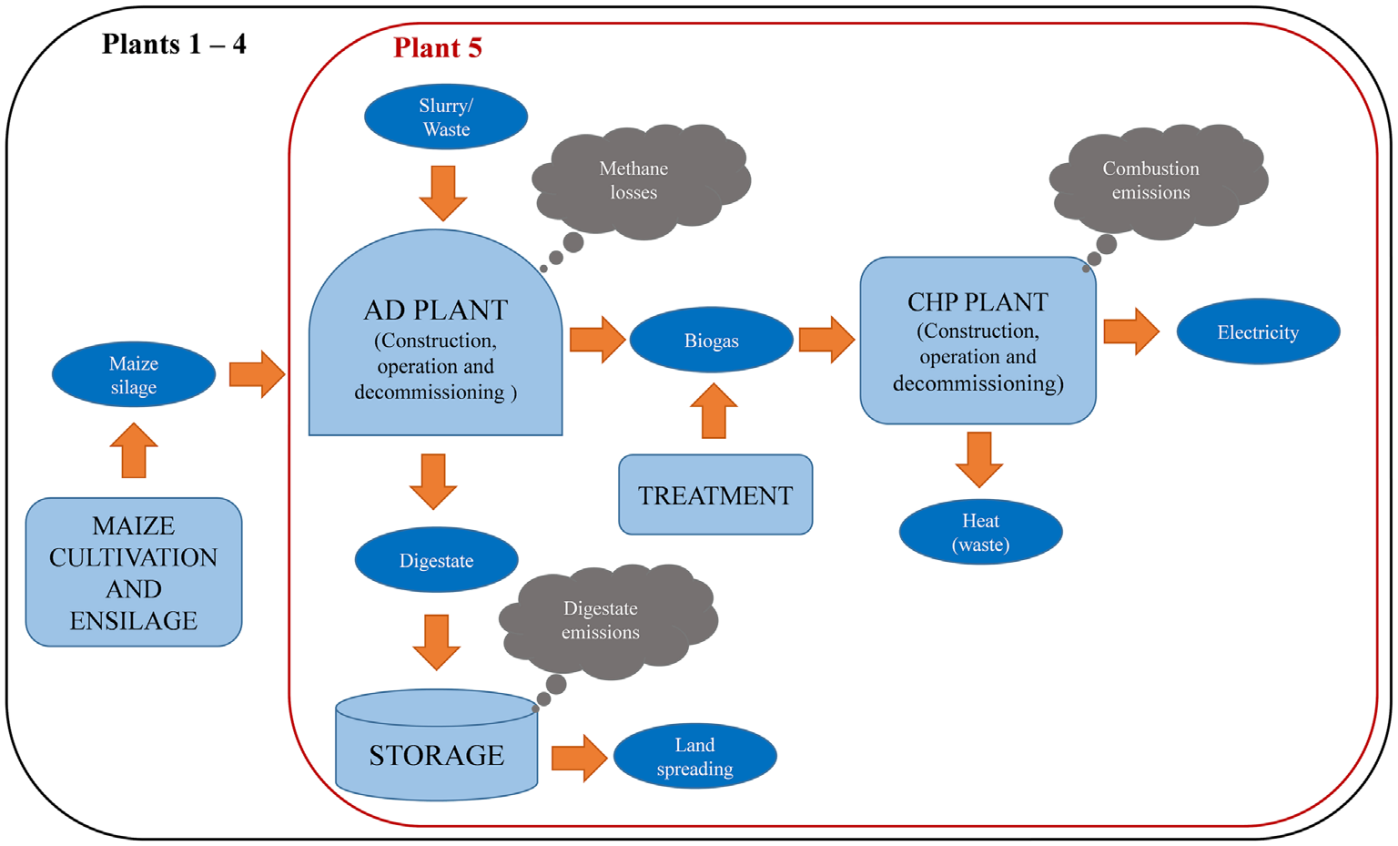
**System boundaries considered in the study**. No environmental impacts are considered for the tomato waste, pig and cow slurry as they are waste. All the impacts are allocated to electricity as heat is not exported from the system.

As indicated in Figure 1, the scope of the study was from “cradle to grave,” including:

- production of maize silage (where used), comprising cultivation, transport from fields to the farm (1 km), and the ensiling;
- collection of slurry and tomato waste and delivery to the AD plants;
- construction and decommissioning of AD and CHP plants;
- production of biogas in the AD plants and its treatment (filtration, dehumidification, and desulfurization);
- cogeneration of electricity and heat in the CHP plants; the heat, except that used for heating the digesters, is considered as waste as it is not used;
- storage and subsequent use of digestate as fertilizer; note that all plants but no. 2 use open storage of digestate.

Electricity distribution and consumption were excluded from the system boundary.

The functional unit was defined as “generation of 1 MWh of electricity to be fed into the grid.” Although heat is cogenerated with electricity, all the impacts were allocated to the latter as the excess heat not utilized in the system is discharged as waste.

### Inventory Data

#### Feedstock Production

The inventory data for the production of maize silage are detailed in Tables S1 and S2 in Supplementary Material. As indicated in the tables, data for field operations were collected directly from the farms. The background data were sourced from Ecoinvent (Nemecek and Kägi, 2007) and modified to match the characteristics of the machinery used for maize cultivation in Lombardy, based on information in Bodria et al. (2006). No environmental impacts were considered for tomato waste and slurry as they are waste.

Ammonia and nitrous oxide emissions as well as nitrate leachates from the application of the digestate and urea as fertilizers were estimated according to Brentrup et al. (2000). Phosphate leachates and run-offs were calculated based on Nemecek and Kägi (2007). To estimate pesticide emissions to the environment, several factors need to be considered, such as the way in which a pesticide is applied, the soil type, and the meteorological conditions during application (EMEP/EEA, 2013). However, considerations of these parameters is often impractical in LCA studies due to a lack of detailed data (Milà i Canals, 2007). Thus, pesticide emissions to air, water, and soil were determined in accordance with Margni et al. (2002) and Audsley (1997), assuming the following partitioning of the active pesticide components: 85% of the total amount applied remains in the soil, 5% in the plant, and 10% is emitted into the atmosphere; furthermore, 10% of the applied dose is lost as a run-off from the soil into the water. This method is also recommended for use by Curran (2012) and was applied in some other LCA studies [e.g., Boschiero et al. (2014), Falcone et al. (2015), and Fantin et al. (2015)].

Land use change was not considered as the maize feedstock is grown on land previously used to cultivate cereals.

The transport and packaging of pesticides and fertilizers were not included in the system boundaries because of a lack of data. This is not deemed a limitation as some other studies found that their contribution was insignificant [e.g., Cellura et al. (2012)].

#### AD and CHP Plants

In all the AD plants evaluated in this study, the digestion takes place in continuously stirred reactors under mesophilic conditions at a temperature of 40°C (±0.2°C), which is controlled and monitored continuously. Therefore, the digesters are operated at the top end of the temperature scale, which for mesophilic digestion ranges from 30 to 40°C (Weiland, 2010). The digesters are made from iron-reinforced concrete and have an expanded polyurethane external insulation. The biomass is fed into the digesters every 90 min in small amounts and heated using the heat generated by the adjacent CHP. As indicated in Table 2, the dry matter content in the digester varies from 8.5 to 10.6%, and the organic loading rate from 0.58 to 3.4 kg/day m^3^. The biogas composition is similar across the plants with the methane content ranging from 52 to 56% of the biogas volume.

The biogas is stored on top of the digesters in a gasometer dome with a spherical cap. Before being fed into the CHP plant, the biogas is filtered through a sand filter, dehumidified in a chiller, and then desulfurized using sodium hydroxide (NaOH). NOx emissions are controlled by a catalytic converter. The digestate is pumped from the bottom of the digesters and stored in open tanks in all the plants except for Plant 2, where it is stored in a covered tank.

The biogas is fed into the CHP plant to generate electricity and heat. Electricity is sold to the national grid while the heat is used for heating the digesters and the excess is dissipated by fan-coolers. The electricity consumption for operating the AD plants is sourced from the national grid to ensure continuous operation during the CHP downtimes. The amount of electricity used by the system ranges from 8.5 to 11% of the total electricity generated (Table 2).

Detailed inventory data for the AD and CHP plants can be found in Tables 2 and 3. The operational data (feedstock production, consumption of electricity and heat, electricity generation) were obtained from the owners. Chemical characterization of different types of feedstock and their biogas production potentials were determined by laboratory tests (Fiala, 2012; Negri et al., 2014; Bacenetti et al., 2015) and used to calculate the biogas production by the AD plants. The emissions from the CHP plants were calculated based on NERI (2010). The useful lifetime of the AD plants was assumed to be 20 years (Nemecek and Kägi, 2007). For the CHP plants, the lifespan is shorter, between 8 and 10 years because of the high content of hydrogen sulfide (Fiala, 2012). At the end of a plant’s useful lifetime, its construction materials were assumed to be landfilled, except for plastic materials, which were incinerated; the influence on the impacts of recycling is explored in a sensitivity analysis later in the paper.

The background data on the construction materials, their transport (120 km by rail and 35 km in 20–28 ton trucks) and landfilling were sourced from the Ecoinvent database v2.2 (Ecoinvent, 2010). Since the data for construction materials for the AD and CHP plants in Ecoinvent correspond to a different plant size (300 m^3^ for the AD and 160 kW_el_ for the CHP plants), the environmental impacts from their manufacture were estimated by scaling up or down their capacity to match the sizes of the AD and CHP plants considered in this study. This was carried out following the approach used for cost estimation in scaling up process plants (Coulson et al., 1993) but instead of costs, estimating environmental impacts as follows (Whiting and Azapagic, 2014):

(1) $$E_{2}=E_{1}\cdot {\left(C_{2}/C_{1}\right)}^{0.6}$$

where *E*_2_ environmental impacts of the larger plant (AD or CHP); *E*_1_ environmental impacts of the smaller plant (AD or CHP); *C*_2_ capacity of the larger plant (volume for the AD plant and installed power for the CHP plant); *C*_1_ capacity of the smaller plant (volume for the AD plant and installed power for the CHP plant); 0.6 scaling factor.

##### Digestate Use and Methane Emissions Credits

**Figure 2:**
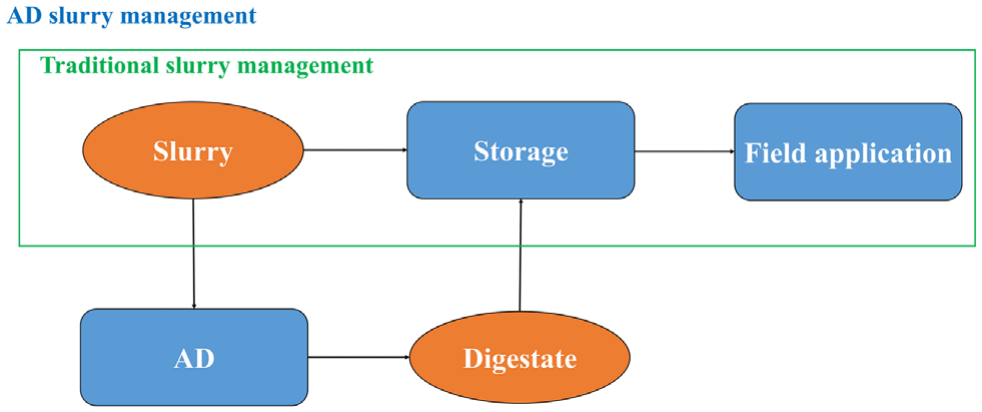
**Traditional and AD slurry management**.

In all the plants except no. 4, the digestate is used as fertilizer on the farms, replacing pig or cow slurries applied previously as part of a traditional slurry management method (see Figure 2). Both digestate and the slurry from Plants 1, 3, and 5 are stored in open tanks before application, during which they emit methane. However, the emissions from digestate are lower than from slurry storage (Amon et al., 2006; Wang et al., 2014), and the AD systems were credited for the avoidance of the emissions. Note that in Plant 2, the digestate is stored in covered tanks, with no emissions of methane (IPCC, 2006); thus, the net emissions from this system are negative (Table 3).

**Table 3:** **Inventory data for the AD and CHP plants (expressed per megawatt hour of electricity)**.

	Unit	Plant 1	Plant 2	Plant 3	Plant 4	Plant 5	Data sources
**AD**							
Pig slurry	ton	8.4	6.0	7.3	–	–	Farm owner
Cow slurry	ton	–	–	–	–	21.0	-||-
Maize silage	ton	0.9	2.25	0.8	2.45	–	-||-
Tomato peel and seeds	ton	1.5	–	–	–	–	-||-
Ear maize silage	ton	–	–	0.66	–	–	-||-
Water	ton	0.94	0.75	–	0.23	–	-||-
Sodium hydroxide	g	28.3	29.6	29.6	29.9	30.0	-||-
Electricity from the grid	MWh	0.09	0.09	0.11	0.09	0.11	-||-
Heat from CHP	MWh	0.42	0.34	0.32	0.38	0.70	-||-
Net biogas production	Nm^3^	280	278	289	252	285	Own calculations based on farm owner’s data
**CHP**							
Electricity generated	MWh	1	1	1	1	1	-||-
Heat generated	MWh	1.3	1.3	1.4	1.1	1.4	Own calculations based on farm owner’s data
**Emissions associated with AD**							
Methane emissions from AD plant	m^3^	3.8	3.8	4.0	3.4	3.9	Bacenetti et al. (2013)
Methane emissions from digestate storage	kg	8.9	0	8.9	8.9	8.9	Edelmann et al. (2011)
Credit for avoiding methane emissions from slurry storage	kg	−6.9	−6.3	−6.0	0	−32.0	Amon et al. (2006) and Wang et al. (2014)
Net emissions of methane	kg	5.9	−2.5	6.9	12.3	−19.2	Own calculations
Ammonia emissions from digestate storage	kg	0.2	0.0	0.2	0.2	0.2	Edelmann et al. (2011)
**Emissions from CHP**							
NOx	g	56.1	56.1	56.1	56.1	56.1	NERI (2010)
NMVOC^a^	g	2.8	2.8	2.8	2.8	2.8	-||-
CH_4_	g	120.6	120.6	120.6	120.6	120.6	-||-
CO	g	86.1	86.1	86.1	86.1	86.1	-||-
N_2_O	mg	444	444	444	444	444	-||-
As	mg	11	11	11	11	11	-||-
Cd	mg	1	1	1	1	1	-||-
Co	mg	58	58	58	58	58	-||-
Cr	mg	50	50	50	50	50	-||-
Cu	mg	86	86	86	86	86	-||-
Hg	mg	33	33	33	33	33	-||-
Mn	mg	53	53	53	53	53	-||-
Ni	mg	64	64	64	64	64	-||-
Pb	mg	1	1	1	1	1	-||-
Sb	mg	33	33	33	33	33	-||-
Se	mg	58	58	58	58	58	-||-
Tl	mg	58	58	58	58	58	-||-
V	mg	11	11	11	11	11	-||-
Zn	mg	1097	1097	1097	1097	1097	-||-

*^a^Non-methane volatile organic compounds*.

**Figure 3:**
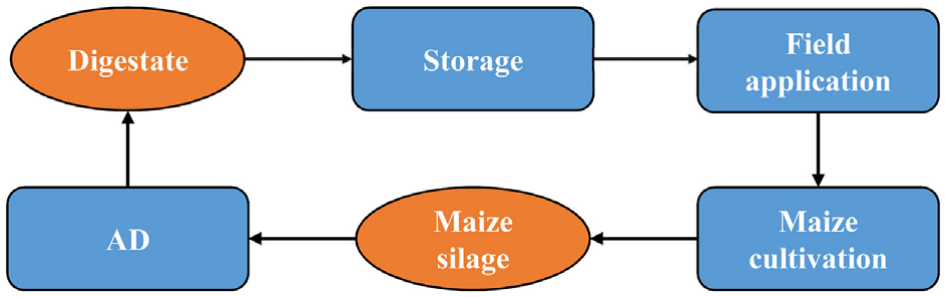
**Maize silage cycle**.

At Plant 4, a closed maize cycle is practiced, whereby the digestate is used as fertilizer for the maize which is fed into the same plant (Figure 3). The digestate at this plant is stored in open tanks.

### Alternative Electricity Sources

Grid electricity was considered here as the main alternative to electricity from biogas. This is due to the latter being fed into the national grid, displacing an equivalent amount of grid electricity. The Italian electricity mix is shown in Figure S1 in Supplementary Material. Given that the electricity mix is dominated by natural gas (53%) (IEA, 2011), biogas electricity was also compared to this option. Furthermore, as biogas is a renewable resource, it was also compared to the other renewables contributing to the Italian mix (see Figure S1 in Supplementary Material). The system boundary for all the alternatives was from “cradle to grave,” and all the data were sourced from Ecoinvent (2010). As for the biogas electricity, distribution and consumption of electricity were not considered.

## Results

**Figure 4:**
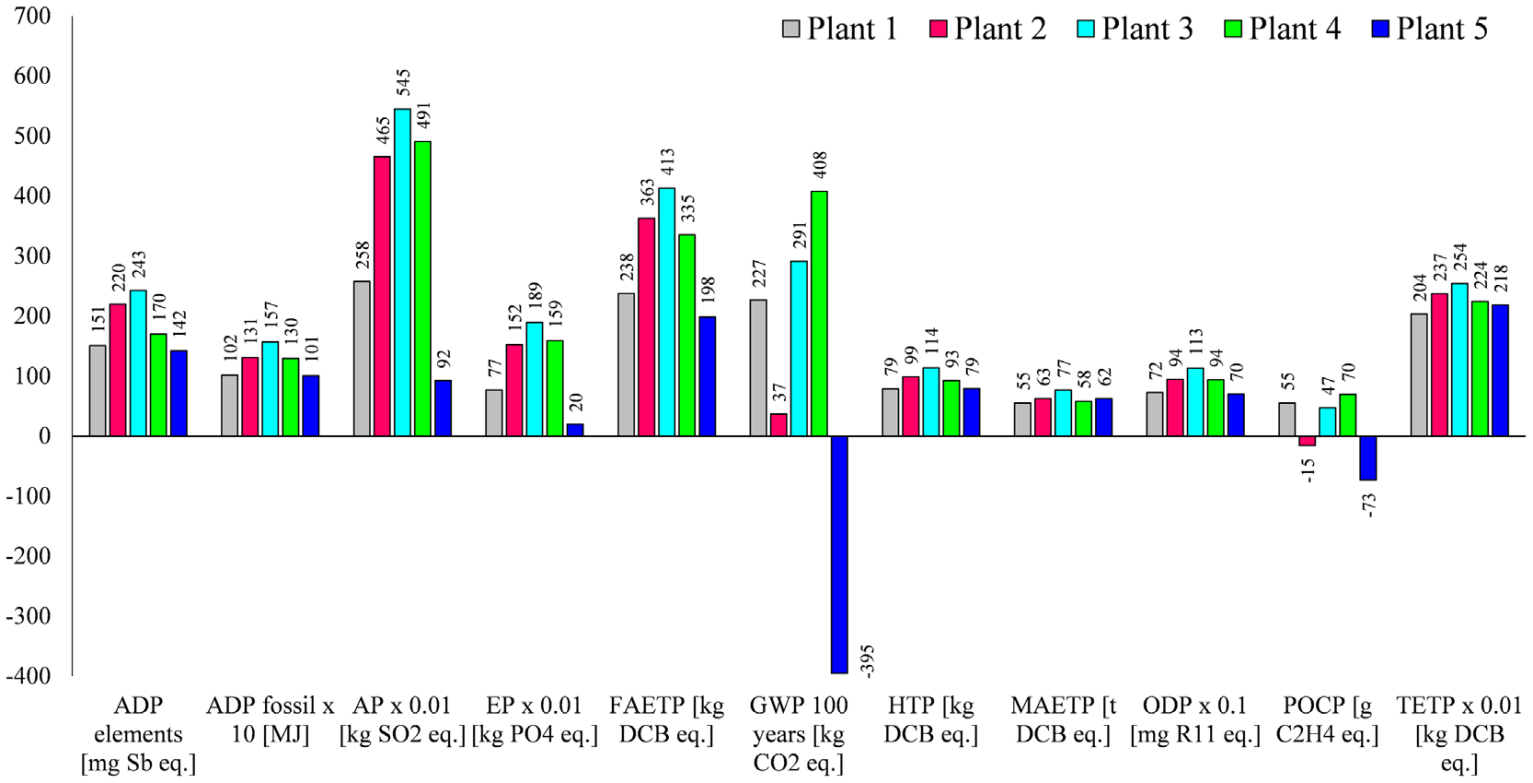
**The environmental impacts associated with the generation of biogas electricity**. All impacts expressed per megawatt hour of electricity generated. Impacts nomenclature: ADP elements, abiotic depletion potential for elements; ADP fossil: abiotic depletion potential for fossil fuels; AP, acidification potential; EP, eutrophication potential; FAETP, freshwater aquatic ecotoxicity potential; GWP, global warming potential; HTP, human toxicity potential; MAETP, marine aquatic ecotoxicity potential; ODP, ozone depletion potential; POCP, photochemical oxidants creation potential; TETP, terrestrial ecotoxicity potential; DCB, dichlorobenzene.

The results suggest that biogas electricity generated by Plant 5 is environmentally the best option among the five plants considered (Figure 4), largely because it does not use maize silage as a feedstock. The exceptions to this are the MAETP and TETP for which Plant 1 is slightly better because these impacts are not affected by maize silage (as discussed further below). Plant 1 is also the second best option for all other impacts apart from GWP and POCP, for which Plant 2 is better because of the lower methane emissions from digestate.

The differences in the impacts for Plants 2 and 4, which are fed with approximately the same amount of maize silage, are due to the differences in the digestate emissions and the capacities of the AD and CHP plants.

Despite the highest biogas production, Plant 3 is the worst option across all the impact categories because of the maize ear silage, which has impacts twice as high as maize silage owing to its lower yield (Table S2 in Supplementary Material). The exceptions to this are GWP and POCP, for which Plant 4 is worst because of the higher net methane emissions (Table 3).

**Figure 5:**
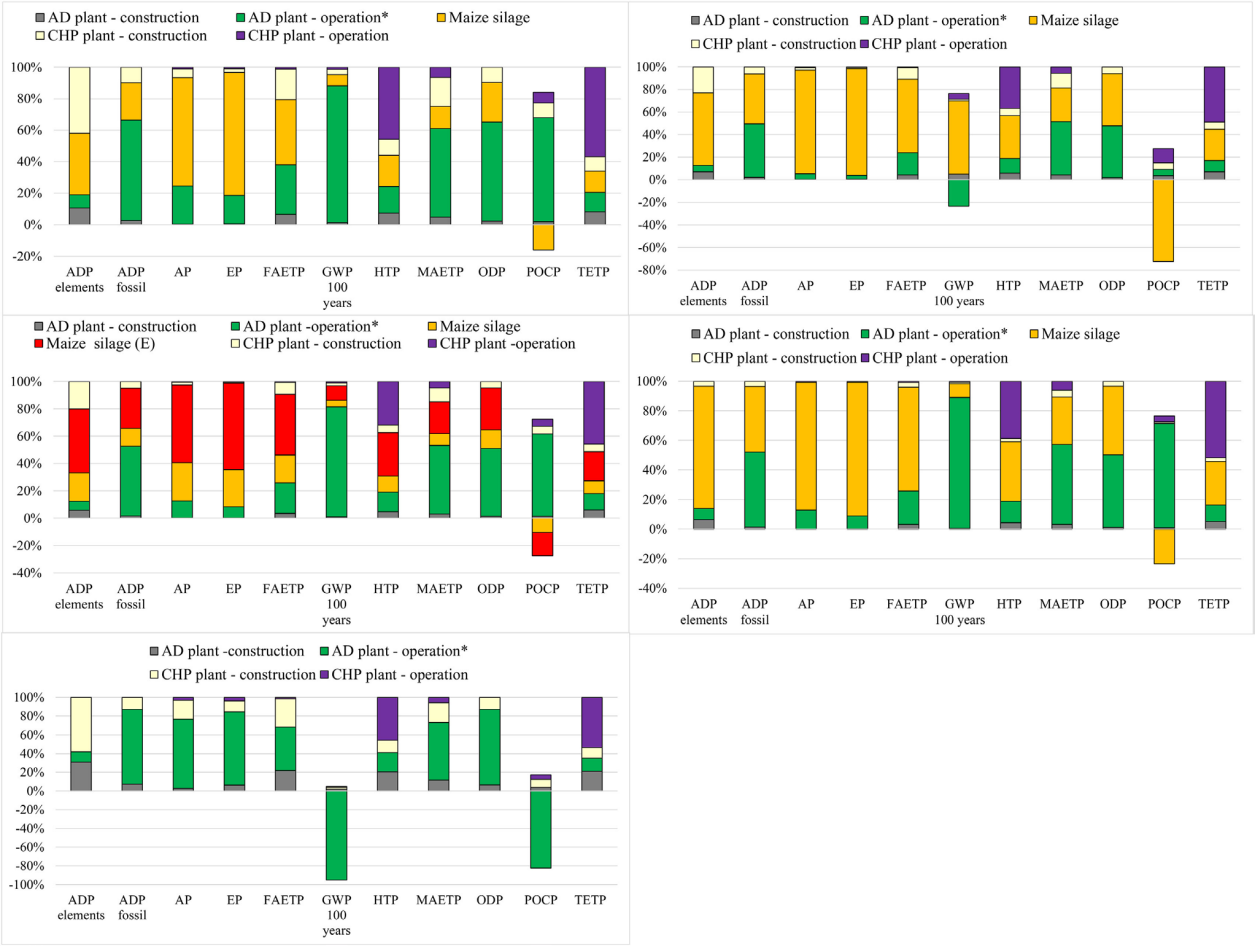
**Contribution analysis for different AD-CHP plants**. **(A)** Plant 1 (top left); **(B)** Plant 2 (top right); **(C)** Plant 3 (middle left); **(D)** Plant 4 (middle right); **(E)** Plant 5 (bottom). AD plant – operation* includes grid electricity used for AD, methane losses during AD and emissions associated with digestate storage. Maize silage **(E)** maize ear silage. For impacts nomenclature, see figure. For the feedstocks, see Table 2. Negative values represent the credits for the avoidance of methane emissions by using digestate as fertilizer instead of animal slurry.

The following sections discuss in more detail the impacts from the different plants (Figure 4) and the contributions of different life cycle stages (Figures 5A–E).

### Abiotic Depletion Potential (ADP Elements and ADP Fossil)

Abiotic depletion of elements and fossil resources range from 142 to 243 mg Sb eq./MWh and from 1010 to 1570 MJ/MWh, respectively, with Plant 5 being the best and Plant 3 the worst option for both impacts.

As indicated in Figures 5A–D, the depletion of elements for Plants 1–4 is mainly due to the cultivation of maize and is associated with the materials used for agricultural machinery. For Plant 5, on the other hand, the major contributors are construction materials for the AD and CHP plants (Figure 5E); the latter is also a hotspot for Plant 1. This is due to economies of scale: they have smaller CHP plants and thus a higher consumption of resources per megawatt hour electricity generated.

As also shown in Figures 5A–D, the major contributors to fossil depletion for Plants 1–4 are the fuel used in the agricultural machinery for maize cultivation and the electricity for the AD plants. For Plant 5, the grid electricity used to operate the AD plant accounts for the majority of this impact (Figure 5E).

### Acidification and Eutrophication Potentials

The estimated AP varies from 2.6 to 5.5 kg SO_2_ eq./MWh and EP from 0.2 to 1.9 kg PO_4_ eq./MWh. As for ADP, biogas electricity generated by Plant 5 is the best and by Plant 3 the worst option for these two impacts. For Plants 1–4, maize cultivation is responsible for the large majority of AP and EP (Figure 5A–D), whereas for Plant 5 (Figure 5E), it is the ammonia emitted during the digestate storage as well as the emissions of acid gases and nutrients in the life cycle of the grid electricity used for AD.

### Global Warming Potential (GWP_100 years_)

The values for GWP range from −395 to 408 kg CO_2_ eq./MWh, with electricity from Plant 5 being the best option and from Plant 4 the worst. The vast majority of GWP (64%) is due to methane emissions from the digestate during its storage. For Plant 2, GWP is mainly from the maize silage (Figure 5B). The negative contributions shown in the figure are due to the methane credits for the avoidance of the traditional slurry management, as described in Section “Digestate Use and Methane Emissions Credits.” For Plant 5, the methane credits are higher than the methane emissions from the digestate, leading to a negative impact of −395 kg CO_2_ eq./MWh (Figure 5E). Note that carbon dioxide emissions from biogas combustion in the CHP plant are not considered as they are biogenic in nature.

### Human Toxicity Potential

This impact is lowest for electricity generated by Plants 1 and 5 [79 kg dichlorobenzene (DCB) eq./MWh] and highest for Plant 3 (114 kg DCB eq./MWh). For Plants 1–4, the main contributor is the production of maize silage and the emissions from biogas combustion, in particular chromium and thallium (see Table 3). For Plant 5, HTP is mainly affected by CHP operation, followed by AD operation and plant construction (Figure 5E).

### Ecotoxicity Potentials (FAETP, MAETP, and TETP)

The lowest FAETP is estimated for Plant 1 (198 kg DCB eq./MWh) and the highest for Plant 3 (413 kg DCB eq./MWh). The production of maize silage and the plant operation are the main contributors to this impact for Plants 1–4. This is mainly due to the emissions of pesticide used for maize cultivation (Table 3) and metals (nickel, beryllium, cobalt, and vanadium) emitted in the life cycle of the grid electricity. It can be noted that Plant 1 has lower MAETP and TETP, which is due to the efficiency associated with economies of scale as these impacts are mainly influenced by the plant operation (Figures 5A,E).

Unlike HTP, the best option for MAETP is Plant 5 at 55 ton DCB eq./MWh but, as for HTP, Plant 3 has the highest impact (77 ton DCB eq./MWh). The main hotspot is grid electricity used for AD because of the emissions of beryllium and hydrogen fluoride in the life cycle of electricity generation.

The same trend is found for TETP, with Plant 5 being the best option (2 kg DCB eq./MWh) and Plant 3 the worst (2.5 kg DCB eq./MWh). Maize silage and CHP operation are the main contributors to TETP for Plants 1–4. Like HTP, the latter is mainly due to the emissions of chromium and thallium from biogas combustion. For Plant 5, CHP operation is the main hotspot (biogas combustion), followed by AD operation and plant construction.

### Ozone Layer Depletion Potential

At 7 mg R11 eq./MWh, Plant 5 has the lowest ODP and, as for most other impacts, Plant 3 the highest (11.3 mg R11 eq./MWh). The main contributors are halons emitted in the life cycle of grid electricity used in AD (related to natural gas transportation), followed by the emissions from diesel used in the machinery during maize cultivation (Plants 1–4).

### Photochemical Oxidants Creation Potential

The POCP ranges from −73 g C_2_H_4_ eq./MWh for Plant 5 to 70 g C_2_H_4_ eq./MWh for Plant 3. For Plants 1, 3, and 4, the impact is largely due to the emissions of methane from the digestate and the methane losses from the AD plant. The negative contributions (Figure 5) are due to two reasons: first, according to the CML 2001 method, nitrogen oxides emitted during the cultivation of maize reduce POCP (Plants 1–4); and second, because of the methane credits (Plant 5).

### Comparison with Alternative Electricity Sources

**Figure 6:**
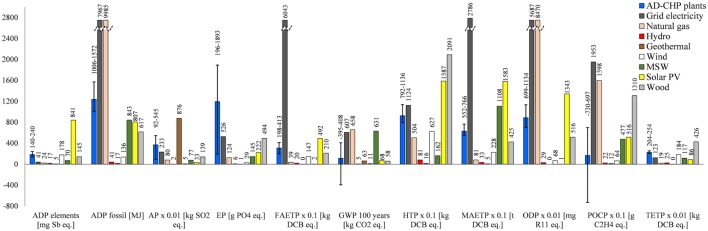
**Comparison of biogas electricity with the alternatives**. All impacts expressed per megawatt hour of electricity. For the AD-CHP plants, the average results are shown, with the error bars representing the impacts ranges for different plants. For impacts nomenclature, see Figure 5. MSW, municipal solid waste; wood, wood chips in a CHP plant.

**Figure 7:**
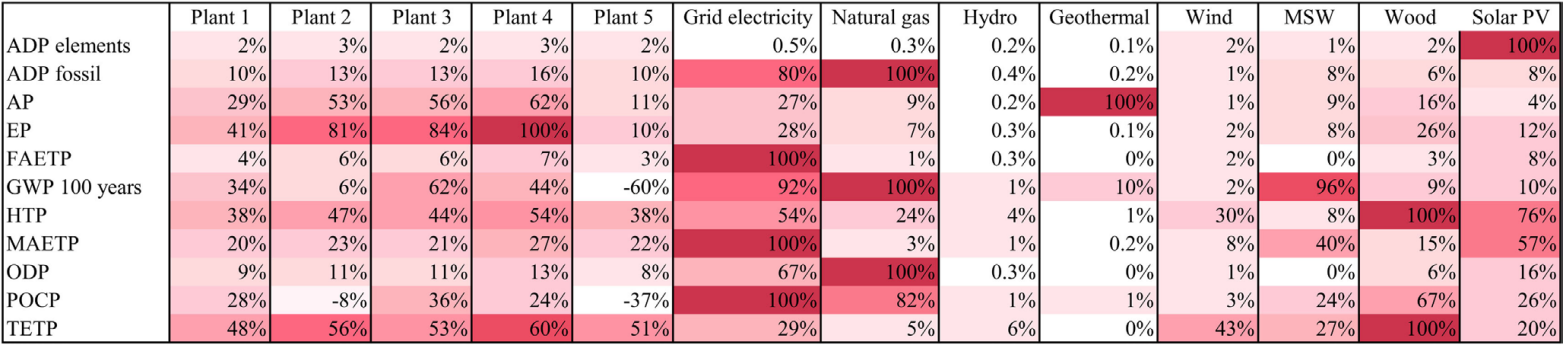
**Heat map of environmental impacts from biogas electricity and the alternatives considered in this study**. The worst option is set at 100% and the others are expressed as a percentage of impact relative to the worst option. Waste, municipal solid waste; MSW, municipal solid waste; wood, wood chips in a CHP plant; solar PV, solar photovoltaics. For impacts nomenclature, see Figure 5.

The biogas electricity is compared to electricity from the grid, natural gas, and renewables in Figure 6 and the ranking of different options with respect to each impact is summarized in the heat map in Figure 7.

As can be seen in Figure 6, grid electricity has higher impacts than electricity from biogas for seven out of 11 categories: ADP fossil, FAETP, GWP, HTP, MAETP, ODP, and POCP. This is mainly due to the high contribution of fossil fuels in the Italian electricity mix. An exception to this is Plant 3 which has a higher HTP than the grid because of the toxic emissions in the life cycle of maize ear silage.

Electricity from the grid also has lower AP (by 10–57%) and EP (32–72%) than biogas electricity; this is due to maize cultivation which contributes significantly to these two impacts (see Figure 5). The exception to this is Plant 5 which has lower impacts than grid electricity (by ~60%) because it does not use maize silage.

Two further impacts are lower for grid electricity: depletion of elements and TETP. This could be explained by the greater economies of scale of the plants on the grid, which require a lower amount of resources and thus have lower toxic emissions on a life cycle basis per unit of electricity generated than the agricultural machinery and the AD-CHP plants.

Unlike grid electricity, electricity from natural gas is environmentally more sustainable than biogas for most categories, except ADP fossil, GWP, ODP, and POCP (Figure 6). In comparison to the renewables, biogas electricity has mostly higher impacts, with a few exceptions. For example, biogas has a lower AP than geothermal power across all the AD-CHP plants considered. Furthermore, Plant 5 has lower GWP and Plant 2 lower POCP than any other renewable option. Biogas is also better than solar PV in terms of ADP elements, HTP, FAETP, MAETP, ODP, and POCP. It also has a lower MAETP than electricity from municipal solid waste and it outperforms wood for HTP, POCP, and TETP.

With a specific reference to GWP, the main driver for biogas production, Plant 5 is the best option overall, sequestering 395 kg CO_2_ eq./MWh. All other plants generate higher GHG emissions than any of the renewable options considered here. The only other impact for which biogas electricity is a better option than any other is POCP, but again only for Plant 5; however, this plant has the highest TETP than any other alternative.

These results are summarized in Figure 7, which shows the percentage difference between the worst option and the rest of the alternatives for each impact. Overall, assuming equal importance of all the impacts, hydropower could be considered the best option and grid electricity the worst, with biogas being on average a middle-ranking option.

### Comparison with Other Studies

As discussed in the Section “Introduction,” comparison of the results from different studies is not easy for the reasons outlined there. The only studies for which comparison is possible are those by Blengini et al. (2011), Dressler et al. (2012), Meyer-Aurich et al. (2012), Bacenetti et al. (2013), Whiting and Azapagic (2014), and Ingrao et al. (2015); for a summary of these studies, see Table 1.

**Figure 8:**
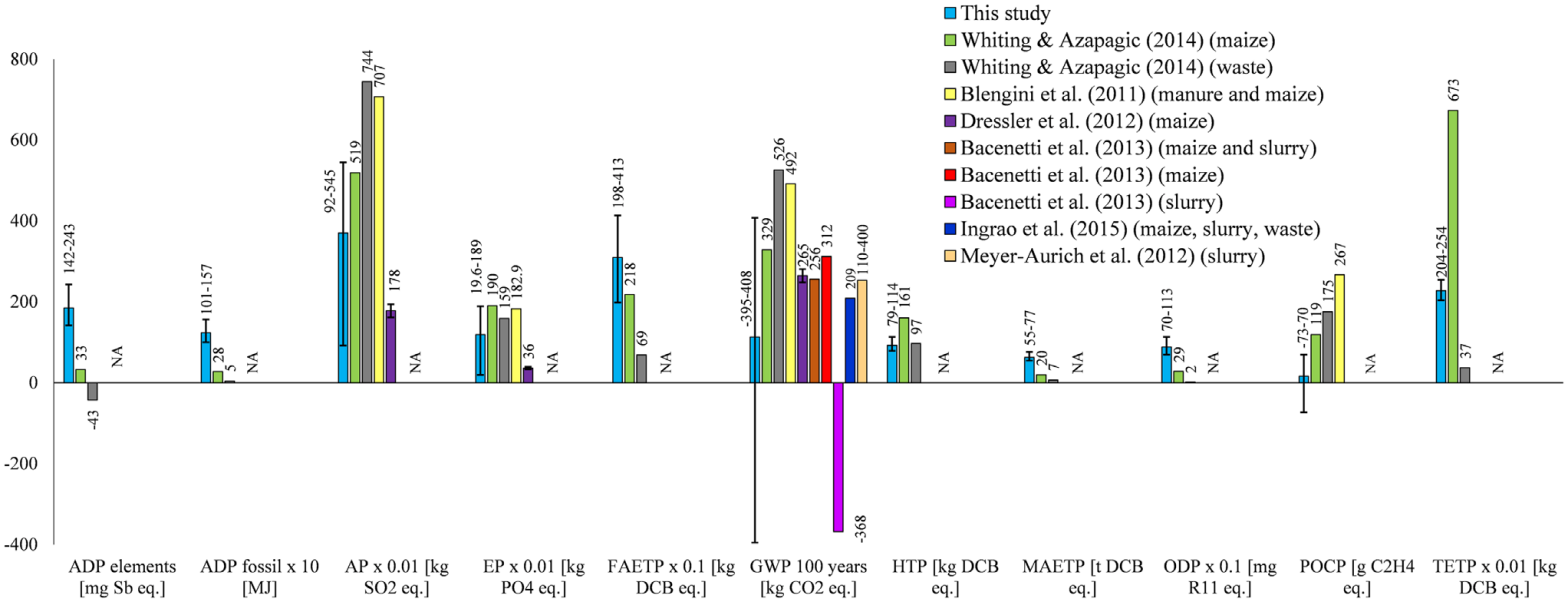
**Comparison of the results with the literature**. All impacts expressed per megawatt hour of electricity. The error bars represent the range of results for the different plants. NA, not available. Waste, agricultural. For impacts nomenclature, see Figure 5.

As can be inferred from Figure 8, the results from the current study compare favorably in terms of AP, EP, GWP, and POCP, given the different assumptions, system credits, and geographical locations across the studies. However, the average GWP estimated in this work appears to be lower than in the other studies, mainly because of Plant 5 which has a negative value for this impact. Nevertheless, the impact for the AD-CHP system using pig slurry reported by Bacenetti et al. (2013) compares well with Plant 5 which uses cow slurry (−368 and −395 kg CO_2_ eq./MWh, respectively). The GWP in Blengini et al. (2011) is consistent with that estimated for Plant 4, while the values found by Dressler et al. (2012), Meyer-Aurich et al. (2012), Bacenetti et al. (2013), and Ingrao et al. (2015) agree well with the results for Plants 1 and 3. It should be noted that, unlike other studies, Meyer-Aurich et al. (2012) have considered land-use change (associated with maize cultivation), finding that it increases GWP by 20%; however, differences in other assumptions cancel out this effect and, consequently, the results still agree with those in the current study.

The comparison of the other impacts is only possible with the study by Whiting and Azapagic (2014), since the other authors did not consider them. As can be seen in Figure 8, the results agree for HTP but differ for ADP, FAETP, MAETP, ODP, and TETP. The reason for these differences could be due to the different updates of the CML method and Gabi software, as well as the different assumptions, credits for fertilizers, and geographical locations. On the other hand, both studies are in agreement that the contribution of the AD and CHP plants construction is significant for ADP elements and the toxicity-related impacts.

### Sensitivity Analysis

Because of their significant contribution to the impacts, the following parameters are considered in the sensitivity analysis:

(i) maize yield;
(ii) heat utilization;
(iii) recycling of AD and CHP construction materials; and
(iv) covered storage of digestate in Plant 4.

The results are discussed in the following sections.

#### Maize Yield

**Figure 9:**
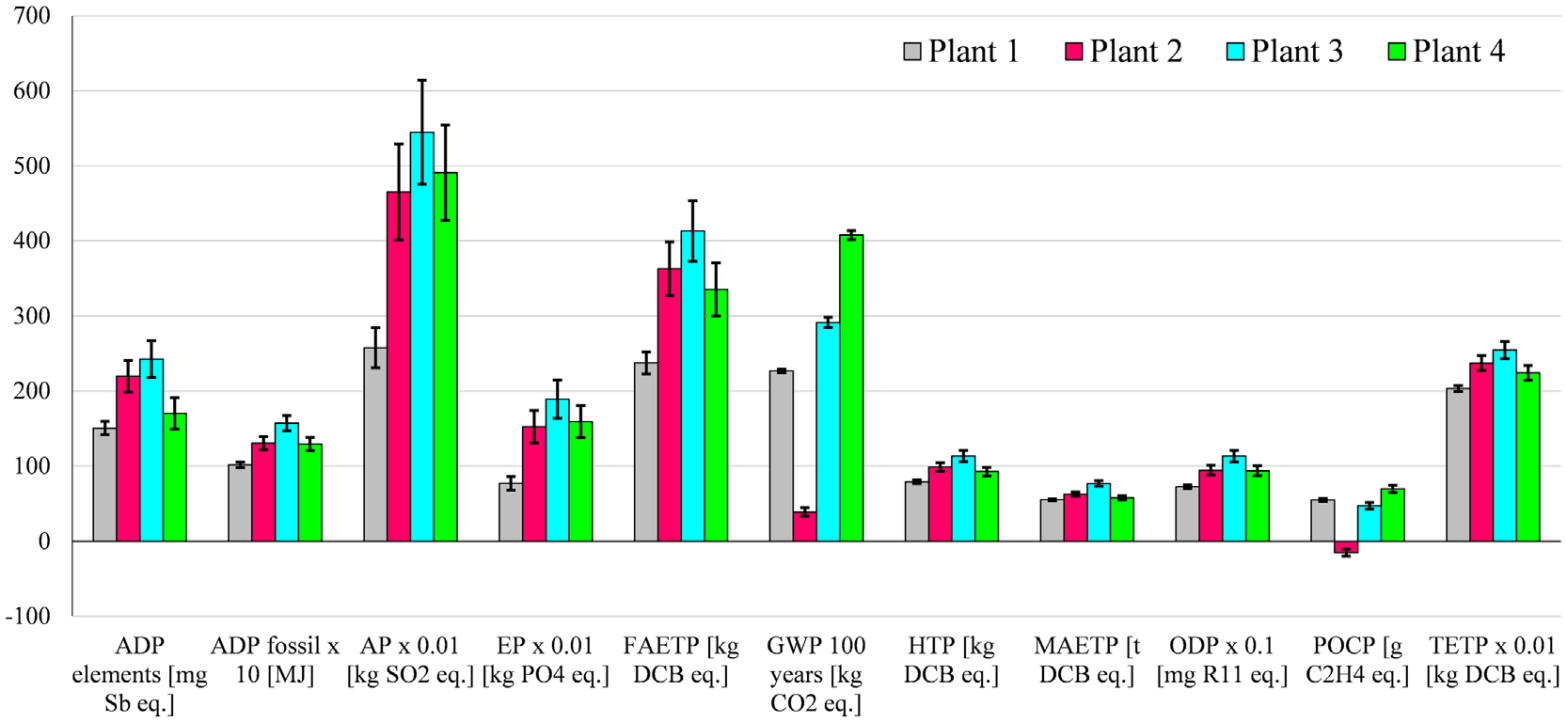
**Sensitivity analysis assuming different maize yields for biogas produced in Plants 1–4**. All impacts expressed per megawatt hour of electricity. The height of the columns corresponds to the yield indicated in Table S2 in Supplementary Material. The error bars refer to the yield variation of ±15%. For impacts nomenclature, see Figure 5.

To explore the effect of this parameter on the impacts, the maize yield was varied by ±15% against the baseline shown in Table S2 in Supplementary Material. The results in Figure 9 suggest that the overall effect of maize yield on the environmental impacts is small for most impacts, except for AP and EP which change by up to 14%. This is to be expected given the high contribution of maize cultivation to these categories.

The ADP elements and FAETP results are also affected for Plant 4, varying by up to 12%, because of the change in the resource requirements for the agricultural machinery and the related toxicity of the construction materials. Despite these changes, the variation in the maize yield considered here does not affect the comparison of biogas with the alternative electricity sources discussed in Section “Comparison with Alternative Electricity Sources.”

#### Heat Utilization

This part of the sensitivity analysis considers a scenario in which the net heat produced by the CHP plants is used instead of being wasted. This is motivated by the introduction of subsidies for heat (see Introduction), which aim to stimulate its utilization. It was assumed that the heat generated by the CHP substitutes a gas boiler for which the AD-CHP systems were credited. The LCA data for the boiler were sourced from Ecoinvent (2010).

**Figure 10:**
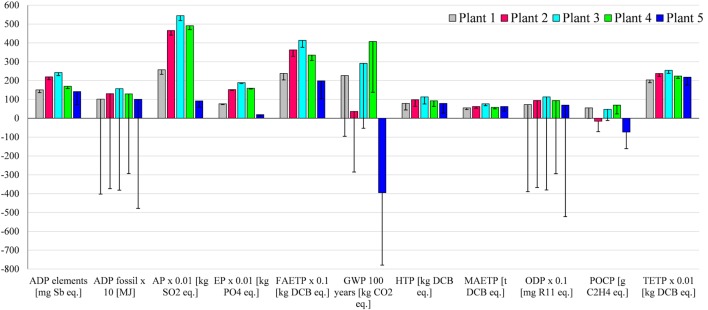
**Sensitivity analysis assuming the net heat produced is used and substitutes a gas boiler**. All impacts expressed per megawatt hour of electricity. Capacity of boiler: >100 kW for Plants 1–4 and <100 kW for Plant 5. For impacts nomenclature, see Figure 5.

As indicated in Figure 10, if the heat were utilized all of the impacts would be reduced, some of them significantly, across the different plants: ADP fossil would be lower by four to six times, GWP up to nine times, ODP by five to eight times, and POCP two to four times. This means that biogas electricity from all five plants would have lower impacts for these categories than any other renewable option considered here. However, there would be no change in ranking with respect to grid electricity because ADP elements, AP, EP, and TETP remain higher for biogas electricity.

#### Recycling of Construction Materials

As mentioned earlier, it was assumed that all the construction materials apart from plastics are landfilled after decommissioning of the plants. Since the construction of the plants has a significant contribution for some impacts, particularly for Plants 1 and 5 (Figures 5A,E), the sensitivity analysis considers if and how they would change if concrete, steel, iron, and platinum (in the CHP catalytic converter) were recycled. For these purposes, the recycling rates for the former three materials were assumed equal to current recycling rates in Italy: 60% for concrete (UNI, 2005) and 74% for steel and iron (Fondazione per lo sviluppo sostenibile, 2012). As there are no data for platinum recycling, a recovery rate of 90% was assumed. Plastic materials were not considered for recycling as their quantity is small.

**Figure 11:**
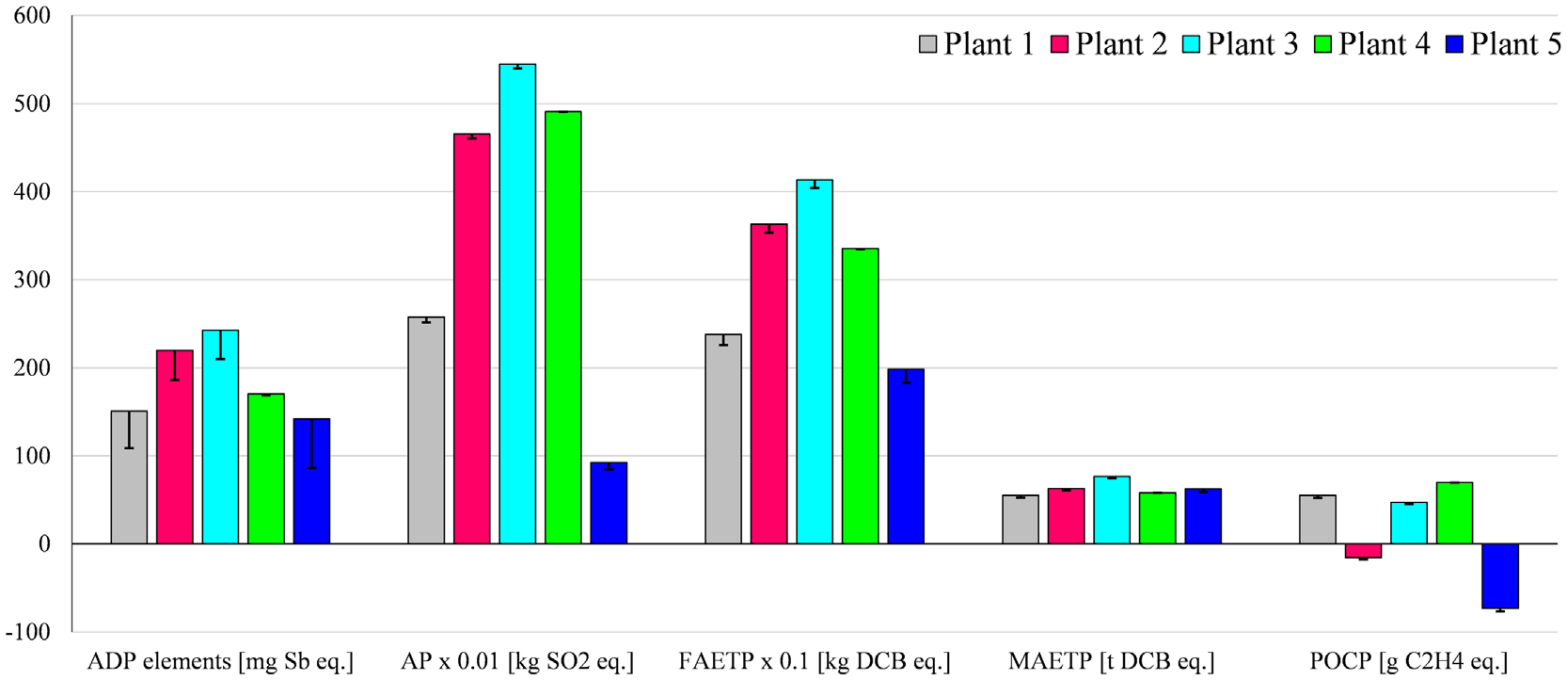
**Sensitivity analysis assuming recycling of construction materials**. For impacts nomenclature, see Figure 5.

The results are presented in Figure 11 for the impacts that are affected by the recycling. The greatest reduction would be achieved for ADP elements (up to 39%) and POCP (up to 13.5%), followed by AP and FAETP (~8%); MAETP would also go down (~5%). The effect on the other impacts is small (<2%).

#### Covered Storage of Digestate

As discussed in Section “Results,” biogas electricity from Plant 4, which uses maize silage as the AD feedstock, has higher GWP and POCP than any other plant. Given that much of that is due to methane emissions from the open storage of digestate (Figure 5D), it is important to consider by how much the impacts would change if the digestate were stored in covered tanks, as in Plant 2.

**Figure 12:**
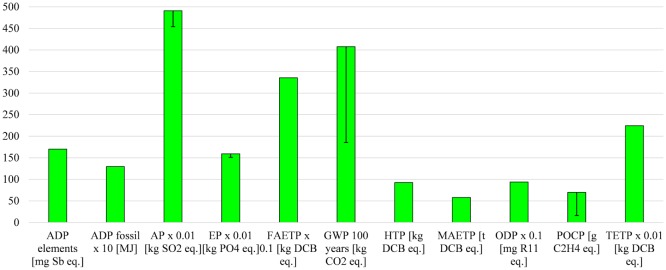
**Sensitivity analysis assuming the covered storage of digestate in Plant 4**. All impacts expressed per megawatt hour of electricity. For impacts nomenclature, see Figure 5.

The results in Figure 12 suggest that both impacts would decrease significantly: GWP by two times and POCP threefold. In that case, Plant 4 would have lower impacts than Plant 1 and 3 but still higher than Plant 2. The AP and EP results would also be reduced, by 7 and 5%, respectively, because of the avoided ammonia emissions. This would make Plant 4 a better option than Plant 2 for these two impacts.

With respect to grid electricity, Plant 4 would have half the GWP. It would also be a better option for POCP with respect to solar PV and waste power plants.

## Conclusion

The aim of this study was to evaluate the life cycle environmental impacts associated with generation of electricity from biogas produced by AD of agricultural products and waste. Five real AD-CHP plants situated in Italy were considered and compared to electricity from the national grid, natural gas, and different renewable technologies.

The results suggest that the main contributors to the impacts from biogas electricity are the production of the maize silage and the operation of the anaerobic digester, including open storage of digestate. Therefore, the system using animal slurry (Plant 5) is the best option among the five plants considered, except for marine and terrestrial ecotoxicity potentials for which the best system is the one utilizing slurry, agricultural waste, and a small amount of maize silage (Plant 1). The plant fed with maize ear silage (Plant 3) is the worst option because of the high impacts of the feedstock, which are almost double that of maize silage.

In reference to the size of AD-CHP plants, larger capacity does not appear to have a positive effect on environmental impacts despite the higher efficiencies typically associated with economies of scale. This is due to the larger plants requiring a high organic load to make them viable, which can only be achieved with cereal feedstocks as they have much higher biogas yield than slurry or agricultural waste. For example, a 1 MW CHP plant requires around 50 ton of maize silage per day but 400–800 ton of slurry. As this amount of slurry cannot be supplied by a single farm, it would have to be collected from different farms and transported to the plant which would not be economically and environmentally viable. Furthermore, the digester would be impractically large (20,000–40,000 m^3^ assuming a hydraulic retention time of 50 days) and thus expensive. Therefore, as the results of this work suggest, it is better to have smaller plants using slurry and waste rather than bigger installations: the latter may be more efficient but require cereal silage, which in turn leads to higher environmental impacts. On the other hand, smaller plants require more resources for construction per unit of electricity generated, so there are some trade-offs.

The results also suggest that utilizing the heat generated by the CHP plant would reduce all the impacts, some of them significantly (specifically depletion of fossil fuels and the ozone layer, global warming, and summer smog), making biogas electricity a better option for these categories than any other renewable alternatives considered here. Recycling the AD and CHP construction materials would reduce the depletion of elements, acidification, freshwater, and marine toxicity as well as summer smog. The latter would also improve in addition to global warming if digestate was stored in covered tanks.

Biogas electricity is environmentally more sustainable than electricity from the grid for seven out of 11 impacts considered. This is due to the high contribution of fossil fuels in the Italian electricity mix. The remaining four impacts, for which grid electricity is a better option, are depletion of elements, acidification, eutrophication, and terrestrial ecotoxicity. Thus, biogas electricity reduces GHG emissions compared to the grid, as intended by government and the European Commission, but aggravates some other impacts.

However, in comparison with natural gas, seven out of 11 impacts are higher for electricity from biogas. It also has mostly higher impacts than the renewables, except for solar PV for which six out of 11 impacts are higher than biogas. Furthermore, biogas is a better option than geothermal power for acidification across all the feedstocks considered. If only slurry is used (Plant 5), it also has lower global warming and summer smog potentials than geothermal. Moreover, marine ecotoxicity is greater for electricity from municipal solid waste than that from biogas.

Focusing on global warming potential which drives biogas production, using slurry as a feedstock (Plant 5) is the best option across all the electricity options considered here, sequestering 395 kg CO_2_ eq./MWh. All the other biogas systems generate higher greenhouse emissions than any of the renewable options considered here. The only other impact for which biogas electricity is a better option than any other is summer smog, but only for the slurry feedstock; however, it also has higher terrestrial ecotoxicity than any other electricity alternative.

In summary, biogas electricity can help reduce GHG emissions relative to fossil-intensive grid electricity such as that of Italy; however, some other impacts are increased. On the other hand, if mitigation of climate change is the main aim, then other renewables have a greater potential to reduce GHG emissions. If, in addition to this, other impacts are considered, then hydro, wind, and geothermal power are better alternatives to biogas. However, if the subsidies for heat utilization are successful, the environmental sustainability of biogas electricity would improve significantly, particularly for global warming, summer smog, and depletion of the ozone layer and abiotic resources. Further policy changes should include a ban on open digestate storage to prevent methane emissions and regulation on digestate spreading on land to minimize emissions of ammonia and related environmental impacts.

Finally, it should be noted that the results obtained in this study correspond to mesophilic digestion at 40°C and may differ from the results for other operating conditions. Furthermore, the analysis did not consider other environmental aspects, such as habitat destruction and biodiversity loss, as they are outside the scope of LCA. These and other impacts could be evaluated in future research alongside economic costs and social impacts as part of a broader sustainability assessment.

## Author Contributions

AA and MF conceived and supervised the work; JB collected the data; AF carried out the LCA study; AA, AF, and JB wrote the paper.

## Conflict of Interest Statement

The authors declare that the research was conducted in the absence of any commercial or financial relationships that could be construed as a potential conflict of interest.
