# Supplementary material for: Life Cycle Environmental Impacts of Electricity from Biogas Produced by Anaerobic Digestion
Source: Front Bioeng Biotechnol. 2016 Mar 11;4:26. doi: 10.3389/fbioe.2016.00026 (PMC4786543; doi:10.3389/fbioe.2016.00026)
Supplement: Supplementary file 1 [file Data_Sheet_1.pdf]

Supplementary Material

Electricity from biogas can help reduce greenhouse gas emissions but other environmental impacts increase

Alessandra Fusi [a], Jacopo Bacenetti [b], Marco Fiala [b], Adisa Azapagic [a]*

*** Correspondence:** adisa.azapagic@manchester.ac.uk

1 Estimation of Life Cycle Environmental Impacts

The life cycle environmental impacts have been estimated using the CML 2001 method (Guineé et al., 2002), April 2013 update, using the equations detailed below (Azapagic, 2011).

1. Abiotic resource depletion potential of elements (ADP elements)

This impact measures depletion of non-renewable resources such as metals and minerals. It is expressed in antimony equivalents and can be calculated using the equation:

$$ADP_{elements} = \sum_{j}^{J} ADP_j \times B_j \quad [1]$$

where:

$ADP_{elements}$ - abiotic resource depletion potential for elements (kg Sb eq.)
ADP_j - abiotic depletion potential for element j (kg Sb eq./kg)
B_j - amount of abiotic resource j used (kg)
J - total number of elements depleted.

2. Abiotic resource depletion potential of fossil fuels (ADP fossil)

ADP fossil measures depletion of fossil energy sources such as coal, oil and natural gas. This impact is expressed in MJ and can be calculated using the equation:

$$ADP_{fossil} = \sum_{j}^{J} ADP_j \times B_j \quad [2]$$

where:

ADP_{fossil} - abiotic resource depletion potential of fossil fuel (MJ)
ADP_j - abiotic depletion potential of fossil fuel j (MJ/kg)
B_j - quantity of fossil fuel (kg)
J - total number of elements depleted.

3. Acidification potential (AP)

The acidification potential is based on the contributions of acidifying pollutants such as sulphur dioxide (SO_2), nitrogen oxides (NO_x), hydrogen chloride (HCl) and ammonia (NH_3) to the potential acid deposition in the form of H^+ ions . It is expressed relative to the AP of SO_2 and can be calculated as:

$$AP = \sum_{j}^{J} AP_j \times B_j \quad [3]$$

where:
AP - acidification potential (kg SO_2 eq.)
AP_j - acidification potential of acid gas j (kg SO_2 eq./kg)
B_j - emission of acid gas j (kg)
J - total number of acid gases.

4. Eutrophication potential (EP)

Eutrophication is the potential of nutrients such as nitrogen (N) and phosphorous (P) to cause overfertilisation of water and soil, which leads to increased growth of biomass (algae). This impact is expressed relative to the EP of PO_4^{3-} and is calculated as:

$$EP = \sum_{j}^{J} EP_j \times B_j \quad [4]$$

where:
EP - eutrophication potential (kg PO_4^{3-} eq.)
EP_j - eutrophication potential of nutrient j (kg PO_4^{3-} eq./kg)
B_j - emission of nutrient j (kg)
J - total number of nutrients.

5. Freshwater aquatic ecotoxicity potential (FAETP)

The freshwater aquatic ecotoxicity potential refers to the impacts of toxic substances on freshwater organisms. It is expressed relative to the FAETP of 1,4-dichlorobenzene (DCB) and estimated by the following formula:

$$FAETP = \sum_{j}^{J} FAETP_j \times B_j \quad [5]$$

where:
FAETP - freshwater aquatic ecotoxicity potential (kg DCB eq.)
$FAETP_j$ - FAETP of substance j (kg DCB eq./kg)
B_j - emission of substance j (kg)
J - total number of substances.

6. Global warming potential (GWP)

Global warming potential expresses the potential of different greenhouse gases (GHGs) to cause climate change. The reference GHG for this impact category is CO_2. The GWP can be calculated as:

$$GWP = \sum_{j}^{J} GWP_j \times B_j \quad [6]$$

where:
GWP - global warming potential (kg CO_2 eq.)
GWP_j - GWP factor for GHG j (kg CO_2 eq./kg)
B_j - emission of GHG j (kg)
J - total number of GHGs.

7. Human toxicity potential (HTP)

The human toxicity potential measures the impact on human health of toxic pollutants such as particles and heavy metals released to air, water and soil. It is expressed relative to the HTP of DCB and be calculated as:

$$HTP = \sum_{j}^{J} HTP_j \times B_j \quad [7]$$

where:
HTP - human toxicity potential (kg DCB eq.)
HTP_j - HTP of substance j (kg DCB eq./kg)
B_j - emission of substance j (kg)
J - total number of substances.

8. Marine aquatic ecotoxicity potential (MAETP)

The MAETP is the impact of toxic substances on marine aquatic ecosystems. Like other toxicity-related impacts, it is also estimated with reference to DCB, as follows:

$$MAETP = \sum_{j}^{J} MAETP_j \times B_j \quad [8]$$

where:
MAETP - marine aquatic ecotoxicity potential (kg DCB eq.)
$MAETP_j$ - MAETP of substance j (kg DCB eq./kg)
B_j - emission of substance j (kg)
J - total number of substances.

9. Ozone layer depletion potential (ODP)

The ODP expresses the potential of substances such as chlorofluorocarbons (CFCs), hydrochlorofluorocarbons (HCFCs) and halons to deplete the stratospheric ozone layer. It is expressed relative to the ODP of CFC-11 and calculated as:

$$ODP = \sum_{j}^{J} ODP_j \times B_j \qquad [9]$$

where:
ODP - ozone depletion potential (kg CFC11 eq.)
ODP_j - ODP of ozone depleting gas j (kg CFC11 eq./kg)
B_j - emission of ozone depleting gas j (kg)
J - total number of ozone depleting gases

10. Photochemical ozone creation potential (POCP)
This impact measures the potential for the formation of photochemical smog, also known as summer smog, due to the reactions of NO_x, volatile organic compounds (VOCs), CH_4 and CO in the presence of sunlight which form oxidants, predominantly tropospheric ozone. This impact is expressed relative to the POCP of ethylene (C_2H_4). It can be calculated according to the equation:

$$POCP = \sum_{j}^{J} POCP_j \times B_j \qquad [10]$$

where:
POCP - photochemical ozone creation potential (kg C_2H_4 eq.)
$POCP_j$ - POCP of substance j (kg C_2H_4 eq./kg)
B_j - emission of substance j (kg)
J - total number of substances.

11. Terrestrial ecotoxicity potential (TETP)
The Terrestrial ecotoxicity potential measures the potential impacts of toxic substances on terrestrial ecosystems. This impact is expressed relative to the TETP of DCB and can be calculated as:

$$TETP = \sum_{j}^{J} TETP_j \times B_j \qquad [11]$$

where:
TETP - terrestrial ecotoxicity potential (kg DCB eq.)
$TETP_j$ - TETP of substance j (kg DCB eq./kg)
B_j - emission of substance j (kg)
J - total number of substances.

2 Supplementary Figures

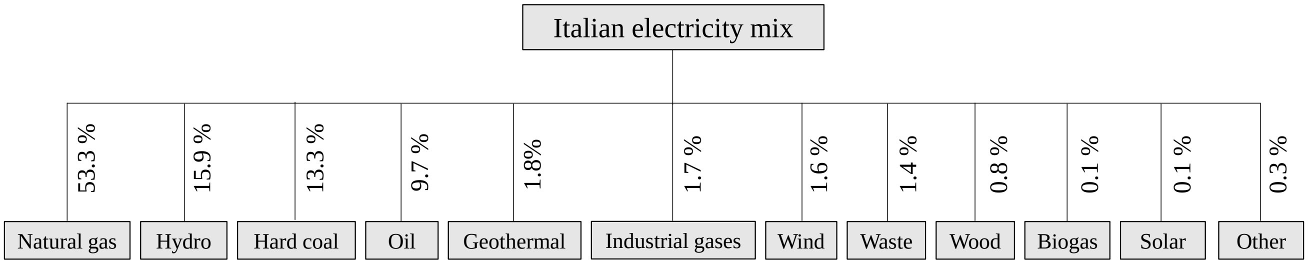

Figure S1. The Italian electricity mix (based on the latest available data from IEA (2011)).

3 Supplementary Tables

Table S1. Agricultural inputs in the production of maize silage (data refer to 1 ha).

Operation	Mass of tractors [a] (kg)	Type, mass [a] and lifetime [b] of other machinery	Time used (hr/ha)	Fuel consumption (kg/ha)	Agricultural inputs
Pre-seeding organic fertilization (digestate)	5050	Slurry spreader<br>1500 kg<br>2000 hr	1.9	3.1	85 t digestate/ha
Ploughing	10,500	Plough<br>2000 kg<br>2000 hr	1.3	22.8	
Harrowing	7300	Rotary harrow<br>1500 kg<br>2000 hr	0.9	23.7	
Sowing	5050	Pneumatic seeder<br>1020 kg<br>2000 hr	0.7	5.7	20 kg seeds/ha
Chemical weeding	4450	Sprayer<br>600 kg<br>1500 hr	0.6	3.1	4 kg lumax [c]/ha<br>2 kg dual [d]/ha
Irrigation	4450	Pump<br>-<br>-	1.2	10.1	4400 m^3/ha
Mechanical weeding	5050	Weeder<br>600 kg<br>1500 hr	0.6	2.9	
Top fertilization	6850	Fertilizer spreader<br>500 kg<br>1500 hr	0.5	2.1	60 kg/ha urea
Harvesting	-	Forage harvester<br>13,500 kg<br>2500 hr	2.2	37.5	
Transport	5050	Farm trailers<br>5000 kg<br>3000 hr	2.2	6.5	
Ensilage	5050	Front loader<br>200 kg<br>2000 hr	0.05[e]	0.44[e]	
Cornstalk chopping[f]	5050	Stalk chopper<br>650 kg<br>2000 hr	1.5	9.5	

[a] Data collected from farms. Lifetime of tractors: 12,000 hr.
[b] Sourced from Bodria et al. (2006).
[c] Active ingredients: atrazine, S-metolachlor, mesotrione.
[d] Active ingredient: S-metolachlor.
[e] The data are expressed per ton of fresh silage.
[f] Applied in the production of maize ear silage.

Table S2. Agricultural outputs in the production of maize silage (data refer to 1 ha).

	Unit	Maize whole plant	Maize ear
Agricultural output [a]			
Maize	t	53.3	21.41
Emissions from application of fertilizers [b]			
Ammonia	kg	54.22	54.22
Nitrogen monoxide	kg	3.64	3.64
Phosphate leaching	kg	0.35	0.35
Phosphate run-off	kg	1.69	1.69
Nitrate leachate	kg	86.3	132.6
Emissions from application of pesticides [c]			
Mesotrione	kg	0.01	0.01
S-metolachlor to air	kg	0.28	0.28
Atrazine to air to air	kg	0.04	0.04
Mesotrione to air	kg	0.01	0.01
S-metolachlor to water	kg	0.28	0.28
Atrazine to water	kg	0.04	0.04
Mesotrione to water	kg	0.01	0.01
S-metolachlor to soil	kg	2.12	2.12
Atrazine to soil	kg	0.33	0.33
Mesotrione to soil	kg	0.09	0.09

[a] Directly measured data.
[b] Own calculation based on Brentrup et al. (2000) and Nemecek and Kägi (2007).
[c] Own calculation based on Margni et al. (2002) and Audsley et al. (1997).

References

Audsley, E. (1997). Harmonization of environmental life cycle assessment for agriculture. European Commission DG VI Agriculture. Silsoe Research Institute, Silsoe.

Azapagic, A., 2011. Assessing environmental sustainability: Life cycle thinking and life cycle assessment. Chapter 3. *In: Sustainable Development in Practice: Case Studies for Engineers and Scientists.* 2nd ed. (Azapagic, A. & Perdan, S., eds.). Chichester: John Wiley & Sons.

Bodria, L., Pellizzi, G., Piccarolo, P. (2006). *Il trattore e le macchine operatric*i. Bologna: Ed. Edagricole.

Brentrup, F., Küsters, J., Lammel, J., Kuhlmann, H. (2000). Methods to estimate on-field nitrogen emissions from crop production as an input to LCA studies in the agricultural sector. *Int. J. Life Cycle Assess*. 5, 349–357.

Guinée, J.B., Gorrée, M., Heijungs, R., Huppes, G., Kleijn, R., Koning, A., et al. (2002). Handbook on life cycle assessment. Operational guide to the ISO standards. I: LCA in perspective. IIa: Guide. IIb: operational annex. III: scientific background. Dordrecht: Kluwer Academic Publishers; 2002.

IEA (2011). OECD - Electricity and heat generation. Electricity information statistics (database). International Energy Agency, 2011. www.oecd-ilibrary.org/energy/data/iea-electricity-information-statistics_elect-data-en.

Margni, M., Rossier, D., Crettaz, P., Jolliet, O. (2002). Life cycle impact assessment of pesticides on human health and ecosystems. *Agr. Ecosyst. Environ*. 93, 379–392.

Nemecek, T., Kägi, T. (2007). Life cycle Inventories of agricultural production systems. Ecoinvent report version 2.0. 2007; Volume: 15. Swiss Centre for LCI, ART. Dübendorf and Zurich, CH.
